# Levels of uPA and PAI-1 in breast cancer and its correlation to Ki67-index and results of a 21-multigene-array

**DOI:** 10.1186/s13000-018-0737-5

**Published:** 2018-08-31

**Authors:** Hans-Ullrich Völker, Michael Weigel, Annette Strehl, Lea Frey

**Affiliations:** 1 Pathology, Leopoldina Krankenhaus GmbH, Gustav-Adolf-Str 8, D-97422 Schweinfurt, Germany; 2 Department of Gynecology, Leopoldina Krankenhaus GmbH, Gustav-Adolf-Str 8, D-97422 Schweinfurt, Germany; 3 Institute for Pathology, University of Wuerzburg, Josef-Schneider-Str. 2, D-97080 Wuerzburg, Germany

**Keywords:** Breast cancer, uPA, PAI-1, Multigene-array, OncotypeDX®

## Abstract

**Background:**

Conventional parameters including Ki67, hormone receptor and Her2/neu status are used for risk stratification for breast cancer. The serine protease urokinase plasminogen activator (uPA) and the plasminogen activator inhibitor type-1 (PAI-1) play an important role in tumour invasion and metastasis. Increased concentrations in tumour tissue are associated with more aggressive potential of the disease. Multigene tests provide detailed insights into tumour biology by simultaneously testing several prognostically relevant genes. With OncotypeDX®, a panel of 21 genes is tested by means of quantitative real-time polymerase chain reaction.

The purpose of this pilot study was to analyse whether a combination of Ki67 and uPA/PAI-1 supplies indications of the result of the multigene test.

**Methods:**

The results of Ki67, uPA/PAI-1 and OncotypeDX® were analysed in 25 breast carcinomas (luminal type, pT1/2, max pN1a, G2). A statistical and descriptive analysis was performed.

**Results:**

With a proliferation index Ki67 of < 14%, the recurrence score (RS) from the multigene test was on average in the *low risk* range, with an intermediate RS usually resulting if Ki67 was > 14%. Not elevated values of uPA and PAI-1 showed a lower rate of proliferation (average 8.5%) than carcinomas with an increase of uPA and/or PAI-1 (average 13.9%); *p* = 0.054, Student’s t-test. When Ki67 was > 14% and uPA and/or PAI-1 was raised, an intermediate RS resulted. These differences were significant when compared to cases with Ki67 < 14% with non-raised uPA/PAI-1 (*p* < 0.03, Student’s t-test). Without taking into account the proliferative activity, an intermediate RS was also verifiable if both uPA and PAI-1 showed raised values.

**Conclusion:**

A combination of the values Ki67 and uPA/PAI-1 tended to depict the RS to be expected. From this it can be deduced that an appropriate analysis of this parameter combination may be undertaken before the multigene test in routine clinical practice. The increasing cost pressure makes it necessary to base the implementation of a multigene test on ancillary variables and to potentially leave it out if not required in the event of a certain constellation of results (Ki67 raised, uPA and PAI-1 raised).

## Background

The risk stratification for breast carcinoma takes into account conventional parameters such as age at onset, menopausal status, tumour size and extension, histological grading and subtype, the assessment of vascular or lymphatic invasion, lympho-nodal status, resection margins and distant metastasis. The determination of the proliferative activity (Ki67) and of the hormone receptor and Her2/neu status are indispensable for prognosis and prediction.

Supplementary analyses of tumour tissue can make the classification more precise. This is especially important in the case of node-negative or minimally positive (max. pN1a) luminal-type carcinomas, because it is in this tumour group that difficulties arise most frequently in making the decision for or against adjuvant chemotherapy.

The serine protease urokinase plasminogen activator (uPA) and the plasminogen activator inhibitor type-1 (PAI-1), as elements of the urokinase plasminogen activator system, are part of the fibrinolytic system [1]. The zymogen plasminogen is converted by uPA into its active, protein-activating and proteolytic form (plasmin) [2]. PAI-1 plays a role in the regulation of the proteolytic activity of uPA [3]. It does not only act as an inhibitor, but also participates in numerous other processes such as cell adhesion and migration, angioneogenesis, signal transduction and apoptosis [4, 5]. uPA and PAI-1 thus play an important role in tumour invasion and metastasis through an interaction with components of the basal membrane, the extracellular matrix and by local proteolysis [6–9].

Increased concentrations of uPA and/or PAI-1 in the tumour tissue of breast carcinomas are associated with a more aggressive progression of the disease, an increased risk of relapse and lower survival rates [10, 11]. Several studies have proved a prognostic significance independent of clinical and histopathological criteria [12–14]. uPA/PAI-1 testing has achieved the level of evidence 1a and has been incorporated into the recommendations of the German Working Group for Gynaecological Oncology (http://www.ago-online.de/de/infothek-fuer-aerzte/leitlinienempfehlungen/mamma; accessed June 4th 2018). This examination is recommended for lymphonodal non-metastasized and Her2/neu-negative breast carcinoma of intermediate grade (G2). In the case of a high protein level of uPA and/or PAI-1 patients could benefit from adjuvant chemotherapy [11].

Various established multigene tests (e.g. OncotypeDX®, Mammaprint®, EndoPredict®, Prosigna®-Assay) supply detailed insights into tumour biology by simultaneously testing several genes that are relevant to prognosis. OncotypeDX® is a 21-gene assay available in Europe since 2009. The standardized and quality-controlled analysis is carried out in a central laboratory in the USA using quantitative real-time polymerase chain reaction (qRT-PCR). A panel of 21 genes is examined, 16 of which are cancer-associated genes as well as five reference genes. The result is used to calculate the numerical recurrence score (RS), which reflects a defined risk of relapse within 10 years from the point of diagnosis. Three risk groups exist for clinical validation: *low risk* (RS < 18), *intermediate risk* (RS 18–30) and *high risk* (RS ≥ 31). The prognostic importance of these groups has been shown by various studies [15–19]. For patients with *high risk* tumours adjuvant chemotherapy has been proven to be useful [20].

However, additional tests also increase the costs of the diagnostic process considerably, and these charges are then not always assumed by German health insurance schemes. The increasing cost pressure represents a substantial problem in clinical practice; responsibilities towards the patient on the one hand and towards the cost bearers on the other hand are often irreconcilable.

Although the prognostic value of common testing procedures is well described in current literature, a correlation of the individual tests amongst each other with an assessment of costs and benefits has not to our knowledge been examined in the researchable literature.

In this initial pilot study, derived from routine clinical work, the intention is to analyse whether the ELISA test for the protein levels of uPA and PAI-1, carried out in addition to conventional histopathological parameters, is similarly suitable for the assessment of prognosis as a multigene test, for which OncotypeDX® has here been selected as an example.

## Methods

Between 2013 and 2016, 954 breast carcinomas were presented and discussed in the interdisciplinary tumour conference of the Breast Cancer Centre at Leopoldina Krankenhaus der Stadt Schweinfurt GmbH (Leopoldina Hospital of the City of Schweinfurt). Male breast carcinomas were excluded. All epidemiological and clinical data of the patients were available as well as the complete postoperative histopathological tumour diagnostics (especially stage, hormone receptor status, Her2/neu status, Ki67). The data set was completely anonymized so that connections to individual cases, particularly patient names and core data, are no longer possible.

Immunohistochemical parameters were determined in a fully-automated device (BondMax, Leica) with a standardized test-kit (Leica).

The commercially available antibody clone 1D5 was used for the oestrogen receptor stain (ER), and the clone *PgR63* (both DAKO®) for the progesterone receptor stain (PR). The immunoreactive score (IRS) was calculated using the method according to Remmele and Stegner, in order to determine the hormone receptor status. This consists of the percentage of positively stained tumour cells and the stain intensity. A score of 0 counts as negative, 1–3 as weakly positive, 4–6 as moderately positive and 8–12 as strongly positive. The Pathology Department of the Leopoldina Krankenhaus Schweinfurt successfully passed the relevant yearly external quality assurance tests (QuIP®) each year within the survey period.

The test for Her2/neu was performed immunohistochemically (clone c-erbB-2, DAKO), and supplemented with a FISH analysis where the result was not clear. The immunohistochemical stains were evaluated in adherence to guidelines with the threshold for a positive result set at 10% tumour cells with a circumferential membranous stain. Here too, the Pathology Department took part successfully in the relevant external quality assurance tests (QuIP®). *The Her2/neu scoring was performed following the current ASCO/CAP-guidelines in accordance with the recently published recommendations* [21]*.*

The Ki67 stain (clone MIB-1, DAKO) was also carried out in a standardized fashion according to protocol. The percentage of tumour cells with immunohistochemical evidence of MIB-1 protein expression was stated as the Ki-67 proliferation index. Here too there had been successful participation in the relevant external quality assurance test (QuIP®).

For 25 carcinomas with primarily surgical treatment (*all of no special type NST*, G2, Elston-Ellis grading), a sample of native tumour tissue was collected to determine the concentrations of uPA and PAI-1. *Because the information from this laboratory test was only relevant for G2 tumors without nodal metastasis in the clinical routine, no carcinomas of other grade or stage (G1 or G3, lymphonodal metastasized) were includable in this study.* From the surgical specimen a sample of frozen tissue was promptly sent to Limbach Laboratory, D-69126 Heidelberg, for an ELISA test. This procedure requires a certain minimum size of tumour (at least 1.3 cm diameter), because on the one hand a tissue sample of around 0.125cm^3^ is required for ELISA testing and on the other hand enough material must be retained to guarantee a complete histopathological analysis despite the removal of tumour tissue. The cut-off value for uPA concentrations which are associated with an increased risk of relapse is ≥3 ng/mg total protein, and that for PAI-1 is ≥14 ng/mg total protein. The results of the external test were available after 5 days on average.

OncotypeDX® testing was also arranged in these cases. For this purpose, a paraffin block containing tumour tissue from the routine appraisal of the surgical specimen was dispatched; the tissue had previously been fixated in 4% buffered formalin. The shipment took place via a specified logistics service provider to a central pathology laboratory (Optipath®) in D-60487 Frankfurt/Main chosen by the provider of the test. From there shipping to the central laboratory in the USA and the reporting back of the test results was organised. On average, the result was available 8 days after the sample was sent.

The analysis was statistically descriptive. *Because most of the evaluated cases showed identical including criteria (G2, N0) no multivariate analysis was performed. The nuclear grade (intermediate or high) and the mitotic count (without exception low) were also highly similar in the 25 cases, so that we found no indication for a separate analysis.*

## Results

For 25 tumours (only luminal-type carcinomas, hormone receptor positive, Her2/neu-negative, G2, pT1 or pT2, not or only minimally nodal metastasized, max. pN1a), the concentrations of uPA and PAI-1 were determined and in addition a multigene test OncotypeDX® was performed. The data for this collective is shown in Table 1.

**Table 1:** Data of the cohort

*n* = 25	
Age	average 52; median 50 (28–71)
pT1	*n* = 12
pT2	*n* = 13
Diameter of tumor	average 2.1 cm; median 2.0 (1.1–5.0)
pN0	*n* = 23
pN1a	*n* = 2 (1/6sn) and (2/24), metastases with max. 0.5 cm diameter
L0	*n* = 15
L1	*n* = 10
V0	*n* = 25
G2	*n* = 25
Hormone receptors for estrogen and/or progesterone	*n* = 25 positive
Her2/neu	*n* = 25 negative (Score 0: *n* = 6, Score 1+: *n* = 18, Score 2 + with negative result in FISH: *n* = 1)

The protein levels of uPA/PAI-1 and the numerical recurrence score (RS) from the multigene test in the groups with the other histopathological parameters (pT1, pT2, lymphatic invasion, and proliferation Ki67 < 14% or Ki67 > 14%) are presented in Table 2.

**Table 2:** Protein levels for uPA/PAI-1 (ng/ml) and Recurrence Score (RS) from multigenetest in different variables

	Average	Median	+/− deviation
uPA pT1 (ng/ml)	4.5	3.6	2.5
uPA pT2 (ng/ml)	2.4	2.4	1.7
PAI-1 pT1 (ng/ml)	20.8	17.0	11.5
PAI-1 pT2 (ng/ml)	18.4	15.0	11.5
RS pT1	19.1	17.0	8.7
RS pT2	13.1	11.0	6.5
uPA L0 (ng/ml)	3.4	2.9	2.8
uPA L1 (ng/ml)	3.6	3.1	1.7
PAI-1 L0 (ng/ml)	22.0	20.0	11.8
PAI-1 L1 (ng/ml)	15.3	11.0	9.5
RS L0	16.9	16.0	9.2
RS L1	14.6	14.0	6.2
uPA Ki < 14 (ng/ml)	3.2	3.5	2.0
uPA Ki > 14 (ng/ml)	3.6	2.8	3.0
PAI-1 Ki < 14 (ng/ml)	18.9	17.0	11.2
PAI-1 Ki > 14 (ng/ml)	20.9	16.0	12.1
RS Ki < 14	14.5	13.0	5.7
RS Ki > 14	18.1	16.0	11.4

*RS* recurrence score, L0/L1 - without/with lymphangioinvasion

No reliable deduction of the results to be expected from additional molecular tests could be made from the conventional parameters tumour stage or lymphatic invasion. For example, pT2 carcinomas showed a substantially lower RS than pT1 carcinomas. The uPA/PAI-1 protein levels tended to be higher with pT1 tumours when compared to pT2 tumours. Factors such as tumour diameter and age at onset did not correlate with any other of the other parameters (values between *r* = − 0.09 and *r* = − 0.13).

**Fig. 1:**
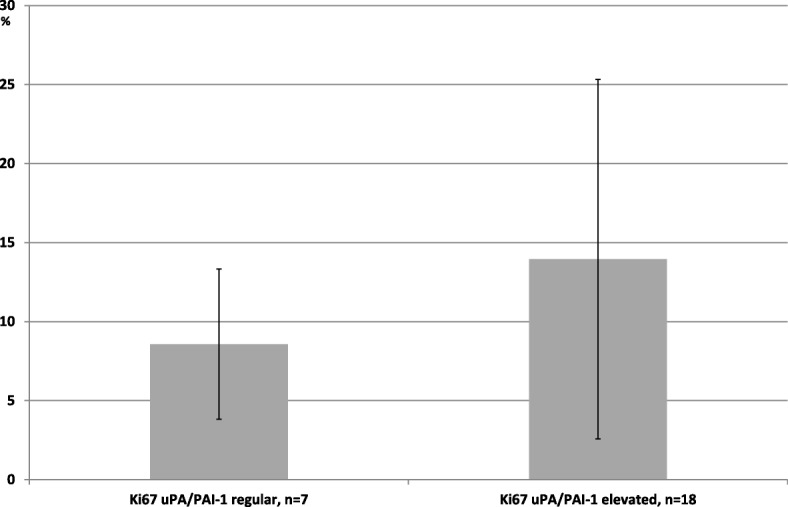
Average of Ki67-Index (MIB-1) in %; breast cancer with regular and elevated uPA and/or PAI-1 (8.5 vs. 13.9%). *p* = 0.054, Student’s t-test

The proliferation index (Ki67 < 14% and > 14%) and RS showed an interdependency. Here on average a *low risk* RS was found with Ki67 < 14% and an intermediate RS with Ki67 > 14% (Table 2). For individual values of uPA or PAI-1 there were no differences in the groups with lower and higher proliferation. In comparison of breast carcinomas with regular values of uPA and PAI-1 to carcinomas with an increase of uPA and/or PAI-1, there was lower rate of proliferation (average 8.5%) in the group with non-increased protein levels than in the group with increased protein level (average 13.9%); *p* = 0.054, Student’s t-test (Fig. 1).

In a Spearman ranking correlation of the variables Ki67, uPA/PAI-1 PAI-1and RS OncotypeDX® there was a trend towards discernible correlations, presented in Table 3.

**Table 3:** Positive correlations between different variables (Spearman correlation)

uPA/PAI-1 to RS OncotypeDX®	0.525
uPA/PAI-1 to proliferative index Ki67	0.460
Ki67 to RS OncotypeDX®	0.517

**Fig. 2:**
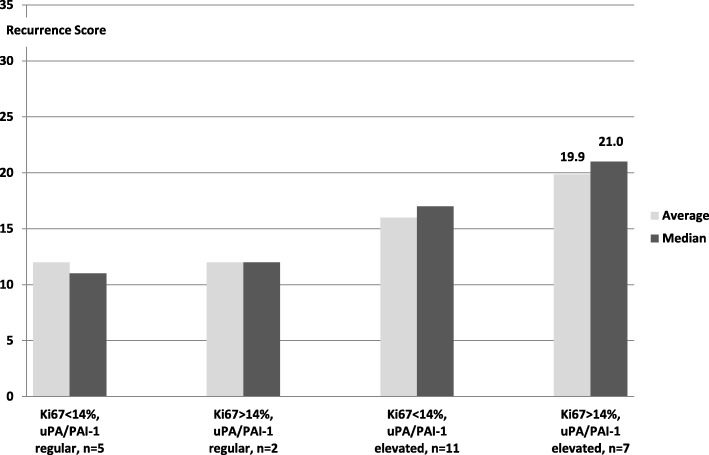
Average and median recurrence scores (RS) of OncotypeDX® depends from results in immunohistochemial measurement of Ki67-index and protein levels of uPA/PAI-1 (Ki67 < 14% and uPA/PAI-1 regular to Ki67 > 14% and uPA/PAI-1 elevated *p* < 0.03)

The differences in the RS became somewhat clearer in sub-group analysis taking into account the proliferation index (threshold 14%) and uPA/PAI-1 status (Fig. 2). With a proliferation of Ki67 > 14% and simultaneous elevation of uPA and/or PAI-1, an average and median RS was observed which already indicates an intermediate risk for a tumour relapse in 10 years (RS > 18). The differences were significant when compared with the group with Ki67 < 14% and non-increased uPA/PAI-1 (*p* < 0.03, Student’s t-test).

**Fig. 3:**
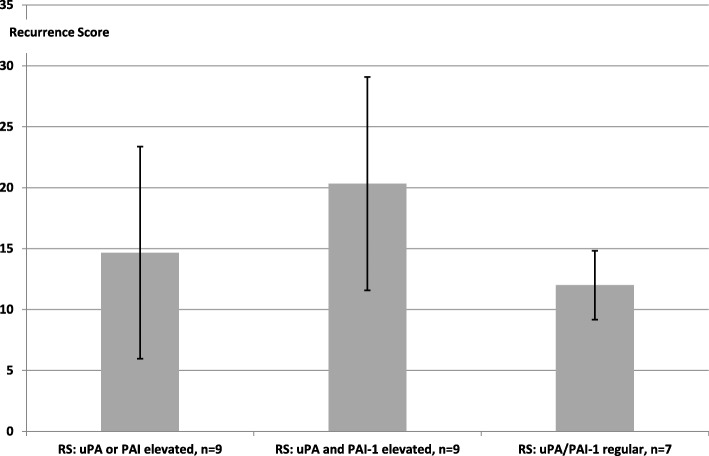
Recurrence score (RS) from OncotypeDX® in elevated uPA or PAI-1, both uPA and PAI-1, and regular values of uPA/PAI-1. The diagram shows the average values, but the median values were on the same level

Without taking into account proliferative activity, an intermediate risk of a tumour relapse (RS > 18) was even then to be observed when both uPA and PAI-1 are increased. If, however, only one of the two parameters was increased, the RS was below the numerical value of 18 as in cases with non-increased uPA/PAI-1 (Fig. 3), *p* = 0.093, Student’s t-test.

**Fig. 4:**
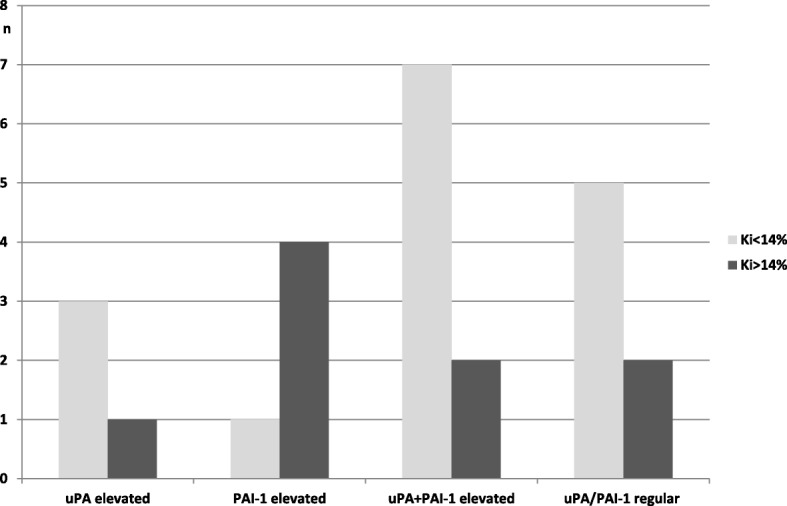
Number of cases with Ki67-Index low (< 14%) and elevated (> 14%) together with results of uPA and PAI-1

With an isolated increase of PAI-1 a trend towards a more frequent high rate of proliferation (Ki67 > 14%) could be observed (Fig. 4).

## Discussion

In this pilot study on a small collection of 25 patients from routine diagnostics, a complete set of tumour biology data, including a protein assay of uPA and PAI-1 and a multigene test (OncotypeDX®), was analysed. A primary expansion of the sample of patients was not achievable from the available resources, but even from the recent results valuable information could be obtained for a more comprehensive study to be planned in the future.

The study showed that with the help of a combination of the values of Ki67 and uPA/PAI-1 the general trend of the recurrence score (RS) to be expected from the multigene test OncotypeDX® can be estimated. Particularly worth emphasising is the evidence of an already elevated recurrence score into the intermediate risk range if Ki67 was > 14% and the protein level of uPA and PAI-1 were increased, and of a RS consistently in the low risk range if both Ki67 and uPA/PAI-1 were not elevated. From this interaction the conclusion may be drawn that in clinical practice an appropriate analysis of this combination of parameters should be envisaged ahead of the multigene test. On the one hand, there are no doubts about the value of multigene tests. On the other hand, however, the costs for these tests are not assumed by some German health insurance schemes. The increasing cost pressure in routine care necessitates making the decision for a multigene test contingent on additional variables and to omit it if not essential in the case of an appropriate constellation of results (Ki67, uPA and PAI-1 all increased or Ki67 and uPA/PAI-1 all not increased). To our knowledge the interrelationships presented here have not yet been examined in the researchable literature. No indications can be derived from the available data as to whether a particular combination of parameters is also reliably associated with a high-risk recurrence score (e.g. by increasing the threshold of the critical Ki67 index from 14 to 20% or 25%). Therefore a further study with a larger patient cohort should be planned. If the result of the pilot study can be confirmed, cost reduction by a factor of 10 could be achieved (according to verbal price information from involved laboratories) with a more targeted use of the multigene tests.

Links are known to exist between the Ki67 index and the values for uPA/PAI-1. Deluche et al. found lower Ki67 indices with negative uPA/PAI-1 than with increased values, with the threshold between low and high Ki67 being set at 20% [22]. They observed that when both parameters were taken into account in therapy planning, 9% fewer patients were received a recommendation for adjuvant chemotherapy than in cases where only the St. Gallen criteria were used. A qualifying comment to be made is that the ELISA (Enzyme-linked Immunosorbent Assay) for uPA/PAI-1 is only possible on fresh or frozen tumour tissue; ideally, a tumour sample of not less than 0.125 cm^3^ must be available. The applicability of the procedure is thus limited by size of the tumour, because experience has shown that with tumour diameters of below 1.3 cm not enough tissue can be obtained without jeopardising the routine diagnostic procedures, especially with regard to the distance of the tumour from the resection margin. Although only an ELISA-based uPA/PAI-1 determination has been validated, efforts are being made to develop immunohistochemical assays for formalin-fixed paraffin-embedded tissue. A study has shown that the uPA/PAI values determined by means of immunohistochemical tests correlate significantly with the values of a validated ELISA [23].

The uPA/PAI-1 test is currently no longer recommended in the new version of the German S3 guideline on breast cancer of 2017. The reason for this was the insufficient data situation for a prognostic assessment, because although the patients included in the older studies were given chemotherapy in the case of increased values, but no anti-hormonal therapy was administered when the values were not increased, so that no direct comparison of the prognosis of the two sample groups could be guaranteed on the basis of present therapeutic standards [24]. Furthermore, the Her2/neu status of the tumours was not known in previous studies. With regard to all breast carcinomas, however, there are indications that a link exists between uPA/PAI-1 and the known intrinsic subtypes. HER2-positive or triple negative carcinomas are much more rarely uPA/PAI-1-negative than luminal-A type carcinomas [25].

According to the guidelines of the *American Society of Clinical Oncology (*ASCO), the levels of uPA and PAI-1 can be drawn upon for the decision for or against adjuvant chemotherapy; this is not recommended for Ki67 alone [26]. The analysis of uPA/PAI-1 can thus usefully supplement the information gained from conventional clinical-pathological parameters in the decision for or against adjuvant chemotherapy in cases of hormone-receptor-positive, Her2/neu-negative breast carcinomas [27], even before a multigene test has to be arranged. *A further aspect is the difference in cost which can be seen as an additional argument in favour of uPA/PAI-1 testing. While this ELISA test tends to cost from 200 to 300 € (in Germany), the multigene array is much more expensive with around 4000 US$. If the uPA/PAI-1/Ki67 constellation points to low or higher risk this can be avoided in selected cases.*

However, no conclusions concerning the success of chemotherapy may be drawn solely from increased values [28]. The risk stratification and therapy planning for a breast carcinoma never take place on the basis of an isolated parameter, not even if a multigene test is available, therefore the above assessment is not problematic.

## Conclusions

The protein-based measurement of uPA/PAI from frozen tumour tissue and additional multigene tests enable a more differentiated risk assessment of the biological tumour behaviour than the sole evaluation of conventional criteria. The decision as to which test procedure is to be used can be made based on the evidence of clinical and methodical validation. In the overall context of the individual disease, extended analyses on tumour tissue must be critically weighed up in view of the benefit to be expected against the arising costs.

## References

[1] Lampelj M, Arko D, Cas-Sikosek N, Kavalar R, Ravnik M, Jezersek-Novakovic B, Dobnik S, Dovnik NF, Takac I (2015). Urokinase plasminogen activator (uPA) and plasminogen activator inhibitor type-1 (PAI-1) in breast cancer - correlation with traditional prognostic factors. Radiol Oncol.

[2] Binder BR, Mihaly J (2008). The plasminogen activator inhibitor “paradox” in cancer. Immunol Lett.

[3] Hildenbrand R, Schaaf A (2009). The urokinase-system in tumor tissue stroma of the breast and breast cancer cell invasion. Int J Oncol.

[4] Andreasen PA, Egelund R, Petersen HH (2000). The plasminogen activation system in tumor growth, invasion, and metastasis. Cell Mol Life Sci.

[5] Chen Y, Kelm RJ, Budd RC, Sobel BE, Schneider DJ (2004). Inhibition of apoptosis and caspase-3 in vascular smooth muscle cells by plasminogen activator inhibitor type-1. J Cell Biochem.

[6] Ulisse S, Baldini E, Sorrenti S, D'Armiento M (2009). The urokinase plasminogen activator system: a target for anti-cancer therapy. Curr Cancer Drug Targets.

[7] Preissner KT, Kanse SM, May AE (2000). Urokinase receptor: a molecular organizer in cellular communication. Curr Opin Cell Biol.

[8] Rosenberg S (2003). The urokinase-type plasminogen activator system in cancer and other pathological conditions: introduction and perspective. Curr Pharm Des.

[9] Reuning U, Magdolen V, Wilhelm O, Fischer K, Lutz V, Graeff H, Schmitt M (1998). Multifunctional potential of the plasminogen activation system in tumor invasion and metastasis (review). Int J Oncol.

[10] Look MP, van Putten WL, Duffy MJ, Harbeck N, Christensen IJ, Thomssen C, Kates R, Spyratos F, Fernö M, Eppenberger-Castori S, Sweep CG, Ulm K, Peyrat JP, Martin PM, Magdelenat H, Brünner N, Duggan C, Lisboa BW, Bendahl PO, Quillien V, Daver A, Ricolleau G, Meijer-van Gelder ME, Manders P, Fiets WE, Blankenstein MA, Broët P, Romain S, Daxenbichler G, Windbichler G, Cufer T, Borstnar S, Kueng W, Beex LV, Klijn JG, O'Higgins N, Eppenberger U, Jänicke F, Schmitt M, Foekens JA (2002). Pooled analysis of prognostic impact of urokinase-type plasminogen activator and its inhibitor PAI-1 in 8377 breast cancer patients. J Natl Cancer Inst.

[11] Janicke F, Schmitt M, Pache L, Ulm K, Harbeck N, Hofler H, Graeff H (1993). Urokinase (uPA) and its inhibitor PAI-1 are strong and independent prognostic factors in node-negative breast cancer. Breast Cancer Res Treat.

[12] Rabi ZA, Todorovic-Rakovic N, Vujasinovic T, Milovanovic J, Nikolic-Vukosavljevic D (2015). Markers of progression and invasion in short term follow up of untreated breast cancer patients. Cancer Biomark.

[13] De Cremoux P, Grandin L, Dieras V, Savignoni A, Degeorges A, Salmon R, Bollet MA, Reyal F, Sigal-Zafrani B, Vincent-Salomon A, Sastre-Garau X, Magdelénat H, Mignot L, Fourquet A, Breast Cancer study Group of the Institut Curie (2009). Urokinase-type plasminogen activator and plasminogen-activator-inhibitor type 1 predict metastases in good prognosis breast cancer patients. Anticancer Res.

[14] Meo S, Dittadi R, Peloso L, Gion M (2004). The prognostic value of vascular endothelial growth factor, urokinase plasminogen activator and plasminogen activator inhibitor-1 in node-negative breast cancer. Int J Biol Markers.

[15] Cronin M, Sangli C, Liu ML, Pho M, Dutta D, Nguyen A, Jeong J, Wu J, Langone KC, Watson D (2007). Analytical validation of the Oncotype DX genomic diagnostic test for recurrence prognosis and therapeutic response prediction in node-negative, estrogen receptor-positive breast cancer. Clin Chem.

[16] Carlson JJ, Roth JA (2013). The impact of the Oncotype DX breast cancer assay in clinical practice: a systematic review and meta-analysis. Breast Cancer Res Treat.

[17] Goldstein LJ, Gray R, Badve S, Childs BH, Yoshizawa C, Rowley S, Shak S, Baehner FL, Ravdin PM, Davidson NE, Sledge GW, Perez EA, Shulman LN, Martino S, Sparano JA (2008). Prognostic utility of the 21-gene assay in hormone receptor-positive operable breast cancer compared with classical clinicopathologic features. J Clin Oncol.

[18] Albain KS, Barlow WE, Shak S, Hortobagyi GN, Livingston RB, Yeh IT, Ravdin P, Bugarini R, Baehner FL, Davidson NE, Sledge GW, Winer EP, Hudis C, Ingle JN, Perez EA, Pritchard KI, Shepherd L, Gralow JR, Yoshizawa C, Allred DC, Osborne CK, Hayes DF, Breast Cancer intergroup of North America (2010). Prognostic and predictive value of the 21-gene recurrence score assay in postmenopausal women with node-positive, oestrogen-receptor-positive breast cancer on chemotherapy: a retrospective analysis of a randomised trial. Lancet Oncol.

[19] Mamounas EP, Tang G, Fisher B, Paik S, Shak S, Costantino JP, Watson D, Geyer CE, Wickerham DL, Wolmark N (2010). Association between the 21-gene recurrence score assay and risk of locoregional recurrence in node-negative, estrogen receptor-positive breast cancer: results from NSABP B-14 and NSABP B-20. J Clin Oncol.

[20] Paik S, Tang G, Shak S, Kim C, Baker J, Kim W, Cronin M, Baehner FL, Watson D, Bryant J, Costantino JP, Geyer CE, Wickerham DL, Wolmark N (2006). Gene expression and benefit of chemotherapy in women with node-negative, estrogen receptor-positive breast cancer. J Clin Oncol.

[21] Wolff AC, Hammond MEH, Allison KH, Harvey BE, Mangu PB, Bartlett JMS, Bilous M, Ellis IO, Fitzgibbons P, Hanna W, Jenkins RB, Press MF, Spears PA, Vance GH, Viale G, McShane LM, Dowsett M. Human epidermal growth factor receptor 2 testing in breast Cancer: American Society of Clinical Oncology/College of American Pathologists Clinical Practice Guideline Focused Update. Arch Pathol Lab Med. 2018; 10.5858/arpa.2018-0902-SA. 10.5858/arpa.2018-0902-SA 29846104

[22] Deluche E, Venat-Bouvet L, Leobon S, Fermeaux V, Mollard J, Saidi N, Jammet I, Aubard Y, Tubiana-Mathieu N (2017). Assessment of Ki67 and uPA/PAI-1 expression in intermediate-risk early stage breast cancers. BMC Cancer.

[23] Lang DS, Heilenkotter U, Schumm W, Behrens O, Simon R, Vollmer E, Goldmann T (2013). Optimized immunohistochemistry in combination with image analysis: a reliable alternative to quantitative ELISA determination of uPA and PAI-1 for routine risk group discrimination in breast cancer. Breast.

[24] Harbeck N, Schmitt M, Meisner C, Friedel C, Untch M, Schmidt M, Sweep CG, Lisboa BW, Lux MP, Beck T, Hasmüller S, Kiechle M, Jänicke F, Thomssen C, Chemo-N 0 Study Group (2013). Ten-year analysis of the prospective multicentre chemo-N0 trial validates American Society of Clinical Oncology (ASCO)-recommended biomarkers uPA and PAI-1 for therapy decision making in node-negative breast cancer patients. Eur J Cancer.

[25] Witzel I, Milde-Langosch K, Schmidt M, Karn T, Becker S, Wirtz R, Rody A, Laakmann E, Schütze D, Jänicke F, Müller V (2014). Role of urokinase plasminogen activator and plasminogen activator inhibitor mRNA expression as prognostic factors in molecular subtypes of breast cancer. Onco Targets Ther.

[26] Harris LN, Ismaila N, McShane LM, Andre F, Collyar DE, Gonzalez-Angulo AM, Hammond EH, Kuderer NM, Liu MC, Mennel RG, Van Poznak C, Bast RC, Hayes DF, American Society of Clinical Oncology (2016). Use of biomarkers to guide decisions on adjuvant systemic therapy for women with early-stage invasive breast Cancer: American Society of Clinical Oncology clinical practice guideline. J Clin Oncol.

[27] Buta M, Džodić R, DJurišić I, Marković I, Vujasinović T, Markićević M, Nikolić-Vukosavljević D (2015). Potential clinical relevance of uPA and PAI-1 levels in node-negative, postmenopausal breast cancer patients bearing histological grade II tumors with ER/PR expression, during an early follow-up. Tumor Biol.

[28] Bellocq JP, Luporsi E, Barrière J, Bonastre J, Chetritt J, Le Corroller AG, de Crémoux P, Fina F, Gauchez AS, Kassab-Chahmi D, Lamy PJ, Martin PM, Mazouni C, Peyrat JP, Romieu G, Verdoni L, Mazeau-Woynar V (2014). uPA/PAI-1, Oncotype DX™, MammaPrint(®). Prognosis and predictive values for clinical utility in breast cancer management. Ann Pathol.

